# ER-α36, a Variant of ER-α, Promotes Tamoxifen Agonist Action in Endometrial Cancer Cells via the MAPK/ERK and PI3K/Akt Pathways

**DOI:** 10.1371/journal.pone.0009013

**Published:** 2010-02-02

**Authors:** Sheng-Li Lin, Li-Ying Yan, Xin-Tian Zhang, Ju Yuan, Mo Li, Jie Qiao, Zhao-Yi Wang, Qing-Yuan Sun

**Affiliations:** 1 State Key Laboratory of Reproductive Biology, Institute of Zoology, Chinese Academy of Sciences and Graduate School, Chinese Academy of Sciences, Beijing, China; 2 Center of Reproductive Medicine, Department of Obstetrics and Gynecology, Peking University Third Hospital, Beijing, China; 3 Department of Medical Microbiology and Immunology, Creighton University Medical School, Omaha, Nebraska, United States of America; Health Canada, Canada

## Abstract

**Background:**

Recently, a novel variant of ER-α, ER-α36 was identified and cloned. ER-α36 lacks intrinsic transcription activity and mainly mediates nongenomic estrogen signaling. Here, we studied the role of nongenomic estrogen signaling pathways mediated by ER-α36 in tamoxifen resistance and agonist action.

**Methodology:**

The cellular localization of ER-α36 was examined by immunofluorescence in MCF-7 cells and Hec1A cells. MCF-7 breast cancer cells, MCF-7 cells expressing recombinant ER-α36 (MCF-7/ER36), Hec1A endometrial cancer cells and Hec1A cells with siRNA knockdown of ER-α36 (Hec1A/RNAiER36) were treated with 17β-estradial (E2) and tamoxifen (TAM) in the absence and presence of kinase inhibitor U0126 and LY294002. We examined phosphorylation of signaling molecules and the expression of c-Myc by immunoblotting, and tumor cell growth by MTT assay.

**Conclusions:**

ER variant ER-α36 enhances TAM agonist activity through activation of the membrane-initiated signaling pathways in endometrial cancer, and that ER-α36 is involved in *de novo* and acquired TAM resistance in breast cancer.

## Introduction

Tamoxifen is a selective estrogen receptor modulator (SERM) with mixed agonist/antagonist activities that has been used widely as an effective treatment of all stages of estrogen receptor (ER)-positive breast cancer [1]. Tamoxifen suppresses the recurrence of breast cancer and reduces the incidence of contralateral breast cancer by 49% [2]. Tamoxifen has also been used as a chemopreventive agent in women who have high risk for breast cancer [3]. It is believed that tamoxifen acts as an antagonist by competing with estrogens for the ligand binding domain of ER, thereby inhibiting ER-mediated mitogenic estrogen signaling [4]. However, the major obstacle to tamoxifen usage is tamoxifen resistance, which occurs *de novo* or can be acquired after its use [5]. In addition, tamoxifen usage increases the incidence of endometrial cancer in postmenopausal women with long-term treatment [6]. The molecular mechanisms underlying both *de novo* and acquired tamoxifen resistance and its agonist action in endometrial tissue are poorly understood.

ER belongs to the steroid hormone family of the nuclear receptor superfamily. It is prevailingly considered that ER acts as a transcription factor that is mainly localized in the cell nucleus [7]. However, accumulating evidence has demonstrated that ER also exists on the plasma membrane and participates in rapid estrogen signaling. It has been reported that ER is modified by posttranslational palmitoylation in the ligand-binding domain that may contribute to its membrane localization [8]. Association of ER and caveolin-1 also was shown to facilitate ER localization on the plasma membrane [9]. Caveolin-1 is a structural protein of caveolae and serves as a scaffold protein to recruit signaling molecules such as growth factor receptors, G proteins, Src family tyrosine kinases and the PI3K [10]. It was postulated that estrogen may rapidly activate different signaling pathways, including MAPK/ERK, phospholipase C, PI3K/Akt and G protein-coupled receptor-activated pathways in the caveolae [11].

Recently, we identified and cloned a novel variant of ER-α with a molecular weight of 36 kDa that was named as ER-α36 [12]. The original 66 kDa ER-α was named ER-α66 [13]. ER-α36 transcript is initiated from a promoter located in the first intron of the ER-α66 gene and is generated from two alternative splicing events. ER-α36 protein thus lacks ligand-dependent and -independent transactivation domain (AF-1 and AF-2), but it retains DNA- binding domain and partial dimerization domain and ligand-binding domains [12]. ER-α36 possesses a unique 27 amino acid domain at the C-terminal that replaces the last 138 amino acids encoded by exons 7 and 8 of ER-α66 gene. Our previous report showed that 17β-estradiol and SERMs such as tamoxifen could induce activation of the MAPK/ERK pathway and stimulate cell proliferation through membrane-associated ER-α36 [14]. We thus hypothesized that ER-α36 may be associated with the agonist activity of tamoxifen. In the present report, we studied the ER-α36 function in ER-positive MCF-7 breast cancer cells and Hec1A endometrial cancer cells, and investigated the contribution of the MAPK/ERK and PI3K/Akt pathways mediated by ER-α36 to the agonist action of tamoxifen in endometrial cancer.

## Results

### ER-α36 Is Expressed on the Plasma Membrane in MCF-7 and Hec1A Cells

**Figure 1:**
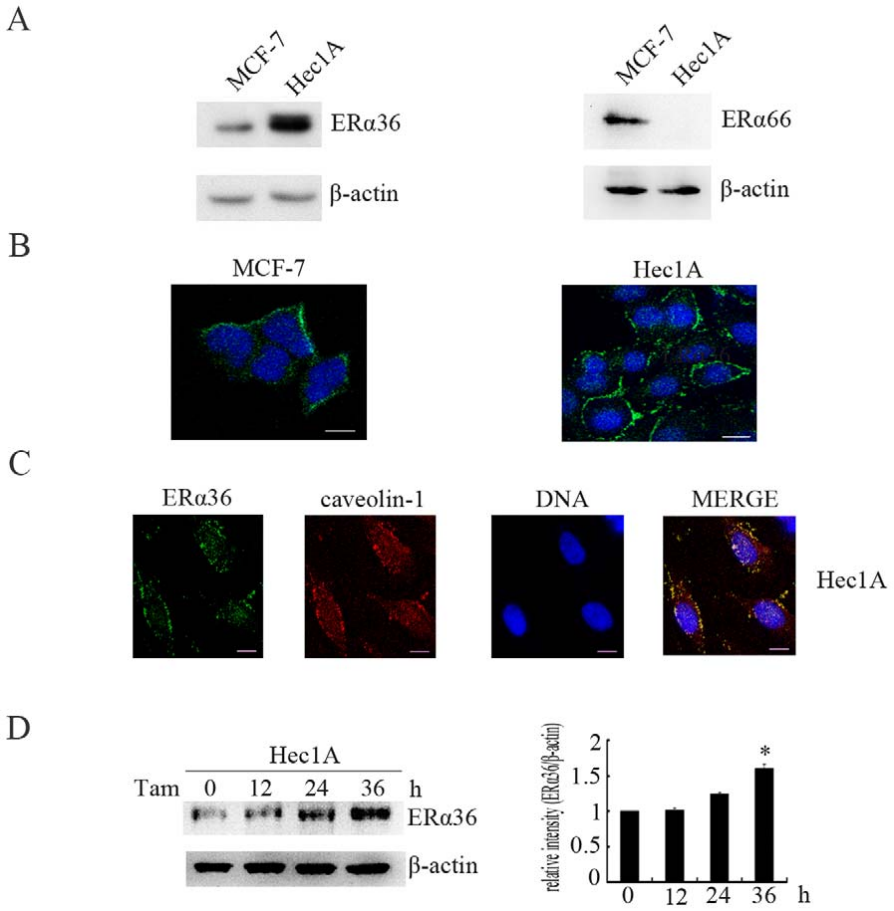
ER-α36 is expressed on the plasma membrane. A, The expression of ER-α36 and ER-α66 protein in MCF-7 and Hec1A cells. Protein extracts were prepared from MCF-7 and Hec1A cells and used for Western blot analysis. B, The localization of ER-α36 in MCF-7 and Hec1A cells. Cells cultured on coverslips were fixed and immunofluorescently stained with a specific anti-ER-α36 antibody (green). The cells were counterstained with Hoechst 33258 (blue). C, The co-localization of ER-α36 and caveolin-1 on the plasma membrane of Hec1A cells. Green: ER-α36; Red: caveolin-1; blue: nuclear; yellow, co-localization signals. Bar, 10 micrometers. D, Time-course analysis of ER-α36 expression in Hec1A cells. Hec1A cells were treated with 2 µM Tam for indicated time points. Levels of protein expression were normalized with β-actin expression level, and each bar represents mean value ± SEM (n = 3). *, P<0.05 compared to untreated cells.

ER-α36 is a novel variant of ER-α66 generated by alternative promoter usage and alternative splicing [12]. To examine ER-α36 expression in MCF-7 cells and Hec1A cells, Western blotting analysis was performed using ER-a36 specific antibody against the unique 20 amino acids at the C-terminal of ER-α36. ER-α36 is expressed in both cell lines (Fig. 1A, left). However, Western blot analysis failed to detect ER-α66 expression in Hec1A cells (Fig. 1A, right), consistent with that Hec1A is an ER-negative cancer cell line [15]. To examine the cellular localization of ER-α36, immunofluorescence assay was performed. In both cell lines, immunofluorescence staining revealed an intense plasma membrane distribution pattern (Fig. 1B). Caveolae are invaginated microstructures on the plasma membrane in which caveolin-1 serves as a scaffold protein to form the signaling complex. As shown in Fig. 1C, caveolin-1 was primarily expressed on the cell surface (red). Merged images of ER-α36 and caveolin-1 showed substantial co-localization signals (yellow) on the plasma membrane.

Next, we analyzed ligand-induced ER-α36 expression. Hec1A cell lines were treated with tamoxifen for different time points and ER-α36 expression was assessed by Western blotting analysis, revealing that ER-α36 expression was increased in tamoxifen treated cells (Fig. 1D).

### ER-α36 Mediates Estrogen- and Tamoxifen- Stimulated ERK Activation

**Figure 2:**
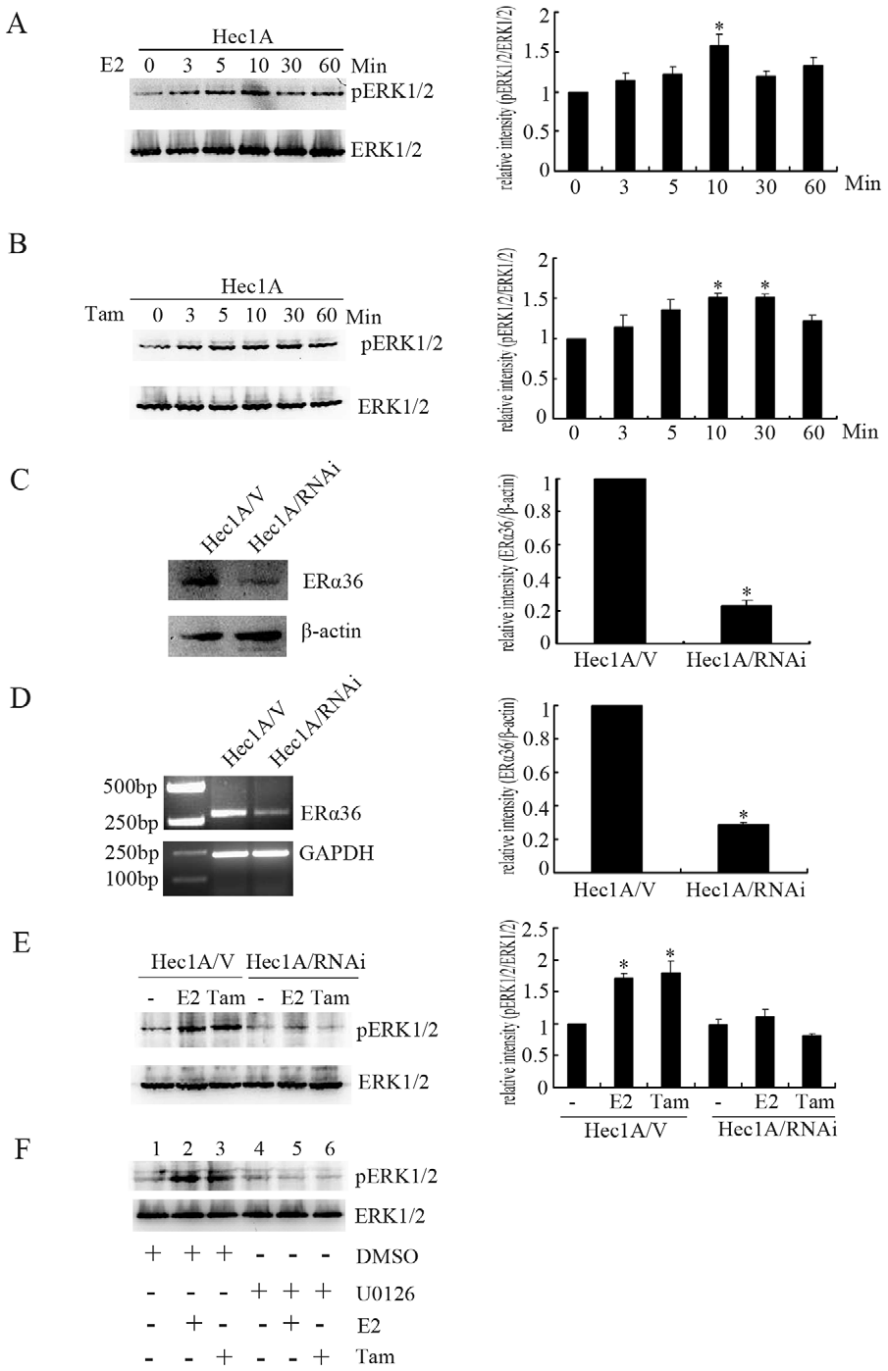
ER-α36 mediates tamoxifen induced activation of the MAPK/ERK in Hec1A cells. A and B, Hec1A cells were treated with 10 nM E2 or 2 µM Tam for the indicated time points. Levels of ERK1/2 phosphorylation were measured in protein extracts with Western blot analysis. Total ERK1/2 was used as loading control. Each bar represents mean value ± SEM (n = 3). *, P<0.05 compared to untreated cells. C and D, ER-α36 expression in Hec1A/V and Hec1A/RNAi cells. Each bar represents mean value ± SEM (n = 3). *, P<0.05 compared to Hec1A/V cells. E, Hec1A/V and Hec1A/RNAi cells treated with 10 nM E2 or 2 µM Tam were analyzed for the levels of ERK1/2 phosphorylation with Western blot. Total ERK1/2 was used as loading control, and each bar represents mean value ± SEM (n = 3). *, P<0.05 compared to untreated cells (−) vs. treatments. F, Lysates from Hec1A cells treated with DMSO (Lanes 1, 2 and 3), 10 nM E2 (Lanes 2 and 5), 2 µM Tam (Lanes 3 and 6) or pretreated with 10 µM U0126 (Lanes 4, 5 and 6) for 30 min were analyzed with Western blot analysis.

To probe the mechanism underlying the agonist effect of tamoxifen in endometrial cancer cells, we decided to examine the function of ER-a36 in tamoxifen treated Hec1A cells. We first examined the phosphorylation levels of MAPK/ERK, a serine-threonine kinase involved in cell proliferation [16]. As shown in Fig. 2A and Fig. 2B, E2 or tamoxifen treatments result in rapid phosphorylation of ERK1/2. Reprobing the membrane with a total ERK1/2 antibody indicated that the total ERK1/2 content was not changed, suggesting that the increased ERK1/2 phosphorylation was not caused by increased ERK1/2 expression.

To test the involvement of ER-α36 in the activites of E2 and tamoxifen observed in Hec1A cells that lack ER-α66 expression, we decided to knock down ER-α36 expression with the siRNA approach. We established stable cell lines that express shRNA expression vector against ER-α36 (Hec1A/RNAi cells) and examined ER-α36 expression (Fig. 2C and 2D). As shown in Fig. 2E, E2 and tamoxifen failed to stimulate phosphorylation of the ERK1/2 in Hec1A cells with ER-a36 knocked down, suggesting that ER-a36 is the receptor that mediates the activities of estrogen and tamoxifen.

Extracellular regulated kinase kinase (MEK) acts upstream of ERK1/2 and could phosphorylate and activate ERK1/2 [17]. The MEK specific inhibitor U0126 effectively inhibited the ERK1/2 activation stimulated by E2 and tamoxifen (Fig. 2F).

**Figure 3:**
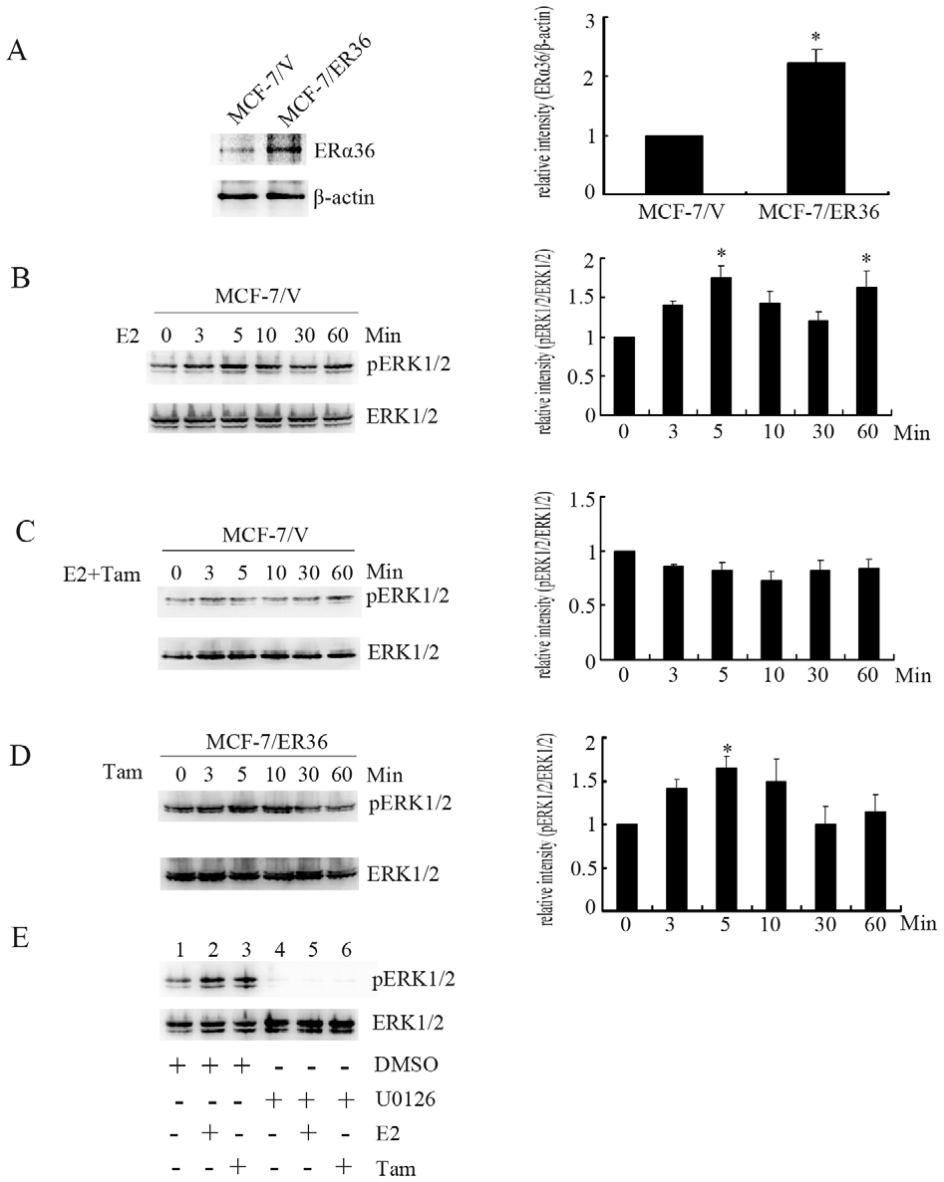
ER-α36 mediates E2 or tamoxifen induced activation of the MAPK/ERK in MCF-7 cells. A, Western blot analysis of ER-α36 expression in MCF-7/V and MCF-7/ER36 cells. Levels of expression were normalized to levels of β-actin, and each bar represents mean value ± SEM (n = 3). *, P<0.05 compared to MCF-7/V cells. B and C, MCF-7/V cells treated with 10 nM E2 alone or with 2 µM Tam together for the indicated time points. Protein extracts were analyzed with Western blot analysis. Total ERK1/2 was used as loading control. Each bar represents mean value ± SEM (n = 3). *, P<0.05 compared to control cells. D, MCF-7/ER36 cells treated with 2 µM Tam for different time points were analyzed for ERK1/2 phosphorylation with Western blot. Levels of expression were normalized to levels of the total ERK1/2, and each bar represents mean value ± SEM (n = 3).*, P<0.05 compared for untreated cells. E, Lysates were prepared from MCF-7/ER36 cells treated with DMSO (Lanes 1, 2 and 3), 10 nM E2 (Lanes 2 and 5), 2 µM of Tam (Lanes 3 and 6) or pretreated with 10 µM U0126 (Lanes 4, 5 and 6) for 30 min and immunoblotted with antibodies against pERK1/2 or total ERK1/2.

We also established stable cell lines from ER-positive MCF-7 breast cancer cells that constitutively express recombinant ER-α36 (MCF-7/ER36 cells) (Fig. 3A). In the control MCF-7 cells transfected with the empty vector, E2 treatment induced phosphorylation of the ERK1/2 (Fig. 3B), which could be abolished by tamoxifen (Fig. 3C). However, tamoxifen induced phosphorylation of the ERK1/2 in MCF-7/ER36 cells (Fig. 3D). MEK specific inhibitor U0126 effectively inhibited the ERK1/2 activation stimulated by E2 and tamoxifen (Fig. 3E). Therefore, these results indicated that ER-α36 mediates the Ras/MEK/ERK pathway induced by both estrogen and tamoxifen and suggested that ER-a36 may be involved in tamoxifen resistance and even promote agonist action of tamoxifen.

### ER-α36 Mediates Estrogen- and Tamoxifen-Stimulated PI3K/Akt Activation

**Figure 4:**
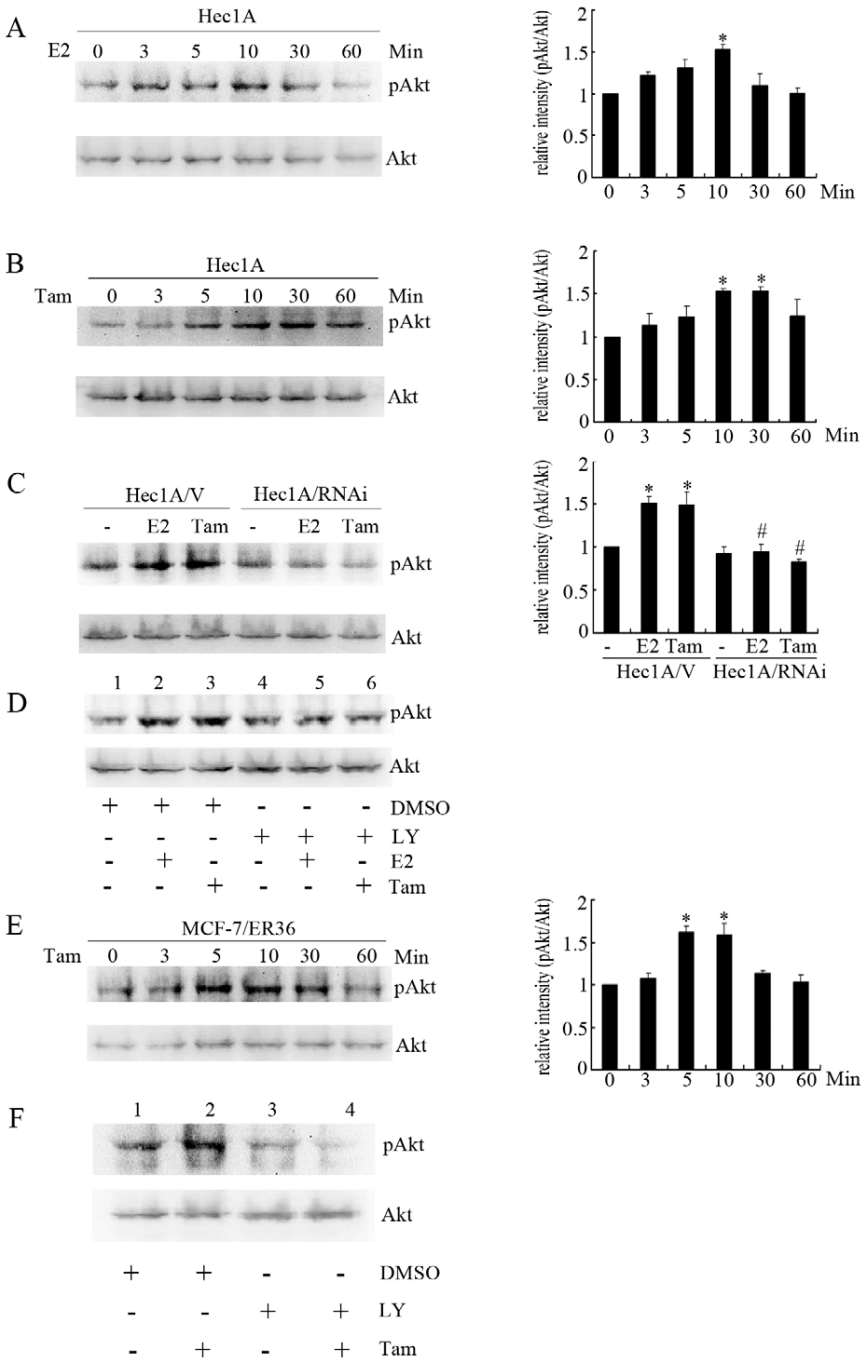
ER-α36 mediates E2 and tamoxifen induced activation of Akt. Hec1A cells were treated with 10 nM E2 (A) or 2 µM Tam (B) for the indicated time points and the lysates were immunoblotted with an antibody against phosphorylated Akt. Levels of phosphorylation were normalized with the total Akt protein, and each bar represents mean value ± SEM (n = 3). *, P<0.05 compared to untreated cells. C, Western blot analysis of the Akt phosphorylation in Hec1A/V and Hec1A/RNAi ER36 cells treated with 10 nM E2 or 2 µM Tam for 10 min. Levels of phosphorylation were normalized with the total Akt protein, and each bar represents mean value ± SEM (n = 3). *, P<0.05 compared to untreated cells. #, P<0.05 compared with E2- or Tam-treated Hec1A/V cells. D, Hec1A cells pretreated with 50 µM PI3K inhibitor LY294002 (LY, Lanes 4, 5 and 6) for 2 h and then treated with 10 nM E2 (Lanes 2 and 5) or 2 µM Tam (Lanes 3 and 6) for 10 min. E, Western blot analysis of Akt phosphorylation in MCF-7/ER36 cells treated with 2 µM Tam for the indicated time points. Expression was normalized to total Akt, and each bar represents mean value ± SEM (n = 3).*, P<0.05 compared for untreated cells. F, Lysates were prepared from MCF-7/ER36 cells treated with DMSO (Lanes 1 and 2), 2 µM of Tam (Lanes 2 and 4) or pretreated with 50 µM PI3K inhibitor LY294002 (LY, Lanes 3 and 4) for 2 h, and immunoblotted with antibodies against phosphorylated Akt or total Akt.

It is well known that the serine/threonine kinase Akt, or protein kinase B, plays an important role in cell proliferation and survival by inhibition of apoptosis [18]. We tested if E2 and tamoxifen treatment also induces activation of the Akt pathway in Hec1A cells. Treatment of E2 and tamoxifen led to rapid phosphorylation of Akt (Fig. 4A and 4B) whereas both E2 and tamoxifen failed to induce the Akt phosphorylation in Hec1A/RNAi cells (Fig. 4C). Tamoxifen also induced Akt phosphorylation in MCF-7 cells that highly express recombinant ER-α36 (Fig. 4E). Pretreatment with the PI3K inhibitor LY294002 abrogated the Akt phosphorylation stimulated by E2 or tamoxifen in both cell lines (Fig. 4D and 4F), indicating that ER-α36 mediates tamoxifen induced Akt phosphorylation through the PI3K pathway in these cells. Thus, our data suggested that ER-α36-mediated activaton of the PI3K/Akt pathway may also be involved in resistance and agonist action of tamoxifen.

### ER-α36 Is Involved in Regulation of c-Myc Protein Expression in Hec1A Cells

**Figure 5:**
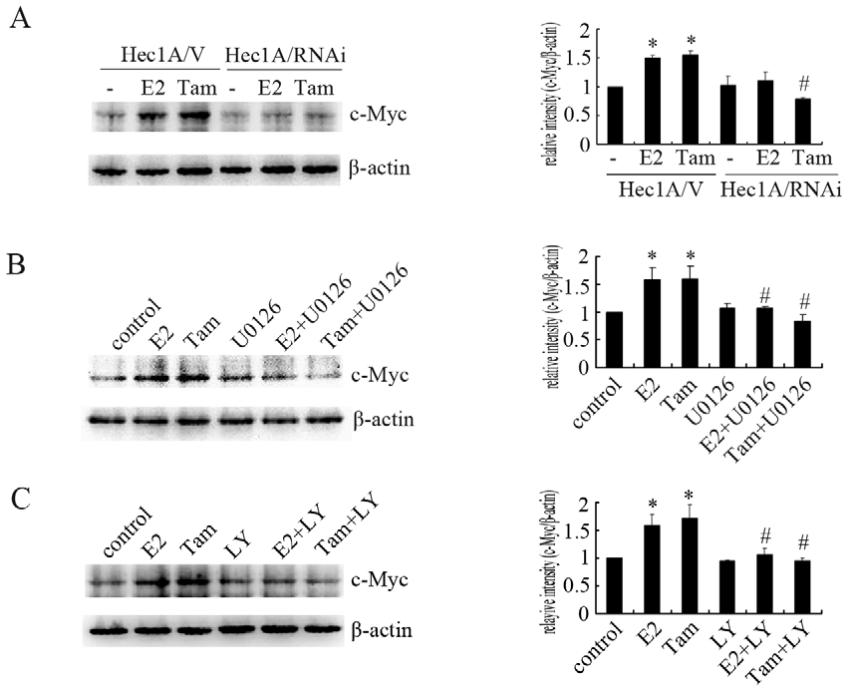
ER-α36 mediates E2 and tamoxifen induced c-Myc expression. A, Western blot analysis of c-Myc expression in Hec1A/V and Hec1A/RNAi cells treated with 10 nM E2 or 2 µM Tam for 12 h. Levels of expression were normalized to the levels of β-actin, and each bar represents mean value ± SEM (n = 3). *, P<0.05 compared to untreated cells (−) vs. treatments. #, P<0.05 compared to Tam-treated Hec1A/V cells. B and C, Hec1A cells were treated for 12 h with 10 nM E2 or 2 µM Tam or together with 10 µM of MEK inhibitor U0126 or 50 µM PI3K inhibitor LY294002. Levels of c-Myc expression were normalized to the levels of β-actin, and each bar represents mean value ± SEM (n = 3). *, P<0.05 compared to untreated cells (−) vs. treatments. #, P<0.05 compared to E2- and Tam-treated Hec1A/V cells.

Protooncogene c-Myc has profound mitogenic effects in cancer cells through its ability to promote cell cycle progression [19]. Antisense oligonucleotides to c-Myc can inhibit breast cancer cells proliferation [20]. Tamoxifen inhibits estrogen-induced c-Myc expression in ER-α66-positive breast cancer cells. However, c-Myc plays an important role in tamoxifen agonist action [21]. We measured the expression levels of c-Myc in Hec1A cells treated with E2 or tamoxifen. As shown in Fig. 5A, treatment with E2 or tamoxifen induced c-Myc expression in Hec1A/V cells but not in Hec1A/RNAi ER-α36 cells, which could be effectively abrogated by the MEK inhibitor U0126 (Fig. 5B) and PI3K inhibitor LY294002 (Fig. 5C).

### ER-α36 Mediates Tamoxifen-Stimulated Cell Proliferation in Hec1A Cells

**Figure 6:**
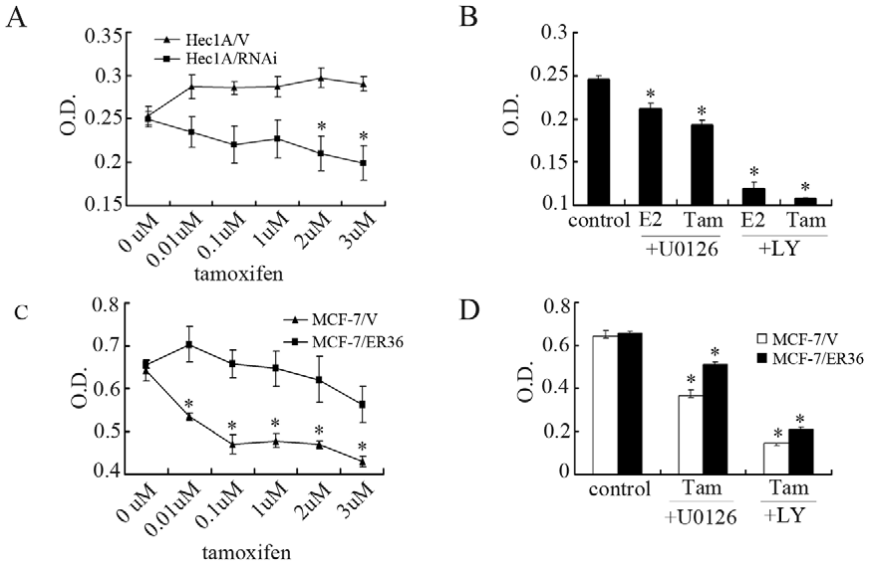
ER-α36 mediates tamoxifen stimulated growth in MCF-7 and Hec1A cells. A, Hec1A cells transfected with the empty expression vector (Hec1A/V) or Hec1A cell lines in which ERα36 had been stably knocked down by shRNA expression (Hec1A/RNAi) were plated 96-well plates (3×10^3^ cells/well). The cells were treated with different concentrations of Tam in medium containing 2.5% dextran charcoal-stripped FBS for 72 h. MTT assay was performed as described in *materials and methods*. Results of three independent experiments were averaged and mean value ± SEM are shown. *, P<0.05 compared to Tam-treated Hec1A/V cells respectively. B, Hec1A cells were treated with 10 nM E2 or 2 µM Tam together with 10 µM of the MEK inhibitor U0126 or 50 µM PI3K inhibitor LY294002 respectively for 72 h, and analyzed by MTT assay. Results of three independent experiments were averaged and mean value ± SEM are shown. *, P<0.05 compared to control cells. C, Empty expression vector transfected MCF-7 cells (MCF-7/V) or MCF-7 cells transfected with ERα36 expression vector (MCF-7/ER36 cells) were plated in 96-well plates (5×10^3^ cells/well). The cells were treated with different concentrations of tamoxifen in medium containing 10% FBS for 72 h. MTT assay was performed. Results of three independent experiments were averaged and mean value ± SEM are shown. *, P<0.05 compared to Tam-treated MCF-7/ER36 cells respectively. D, MCF-7/V and MCF-7/ER36 cells were treated with 2 µM Tam together with 10 µM U0126 or 50 µM PI3K inhibitor LY294002 respectively for 72 h and analyzed by MTT assay. Results of three independent experiments were averaged and mean value ± SEM are shown. *, P<0.05 compared to control MCF-7/V cells.

To further study the role of ER-α36 in tamoxifen agonist activity in endometrial cancer cells, Hec1A/V and Hec1A/RNAi cells were treated with tamoxifen and their prolifaration was measured with the MTT assay. MTT assay showed that tamoxifen stimulated growth of Hec1A/V cells. However, tamoxifen was able to inhibit the growth of Hec1A/RNAi cells in a dose-dependent fashion (Fig. 6A). The cell proliferation induced by tamoxifen was inhibited by the MEK inhibitor U0126 and PI3K inhibitor LY294002 (Fig. 6B), suggesting that both the MAPK/ERK and PI3K/Akt pathways were involved in E2 and tamoxifen stimulated cell growth in endometrial cancer cells.

We observed that tamoxifen strongly inhibited cell proliferation in the MCF-7/V cells, consistent with previous reports that tamoxifen functions as a potent antagonist in ER-positive breast cancer MCF-7 cells [22]. However, MCF-7/ER36 cells that constitutively express high levels of recombinant ER-α36 exhibited insensitivity to tamoxifen treatment (Fig. 6C). The MEK inhibitor U0126 and PI3K inhibitor LY294002 furthermore inhibited growth of both cell lines (Fig. 6D). These results again suggest that high level of ER-α36 expression may confer resistance to tamoxifen.

## Discussion

Tamoxifen is a SERM that has been widely used to treat advanced ER-positive breast cancer and to prevent breast cancer in high risk pre- and post-menopausal women as a chemopreventive agent [23], [24]. However, tamoxifen also has partial estrogenic activity in the uterus that can lead to endometrial hyperplasia [25]. Long-term tamoxifen usage is associated with an increased incidence of endometrial cancer [26]. Here we reported that a novel variant of ER-α, ER-α36, that is highly expressed on the plasma membrane of Hec1A endometrial cancer cells and in the endometrial cancer specimens from patients who had been treated with tamoxifen for at least three years. Both E2 and tamoxifen induced cell proliferation of Hec1A cells presumably through the ER-α36 mediated non-genomic signaling pathways.

A number of hypotheses have been postulated to explain tamoxifen's agonist action in endometrial carcinogenesis. It has been suggested that reactive metabolites of tamoxifen form DNA-adducts and generate mutagenicity in the endometrial tissue [27]. It has also been demonstrated that the AF1 domain of ER-α66 as well as cell- and promoter-specific coactivator recruitment are involved in the tamoxifen agonist action [28], [29]. The role of tamoxifen in endometrial carcinogenesis may utilize distinct genomic activity [30]. Recently, accumulating evidence suggested that membrane-initiated signaling pathways confer tamoxifen resistance and agonist action through different kinase cascades and distinct second messengers [15].

The MAPK family consists of ERK, JNK and P38. ERK plays an essential role in cell growth and proliferation. JNK and P38 are involved in cell differentiation and apoptosis induced by stress stimuli such as UV light [31], γ radiation [32], [33], DNA-damaging and chemopreventive drugs [34]. Many oncogenic signaling molecules activate the MAPK/ERK pathway [35]. ERK expression is usually increased and its activity is up-regulated in breast cancer tissues compared to neighboring normal tissues [36]. Furthermore, tamoxifen resistance *in vivo* is predominantly mediated by non-genomic mechanisms. Genomic estrogen action seems less active [37], [38]. In this study, we found that ER-α36 mediates both E2- and tamoxifen-induced activation of the MAPK/ERK pathway and ER-α36 overexpression in tamoxifen sensitive MCF-7 cells reduced sensitivity to tamoxifen. In addition, ER-α36 mediates tamoxifen induced activation of the MAPK/ERK pathway and also contributes to agonist action of tamoxifen in Hec1A endometrial cancer cells. Endometrial cancer tissues that highly express ER-α36 also displayed high levels of the ERK phosphorylation.

The PI3K/Akt pathway plays an important role in cell growth and survival [39]. Akt is activated by many signaling pathways, such as overexpression of growth factor receptors, [40]. Introduction of a constitutively active Akt into MCF-7 cells could induce tamoxifen resistance by protecting cells from tamoxifen-induced apoptosis [41]. In addition, the Akt activity is dramaticaly increased in tamoxifen- resistant MCF7 cells [18]. In phosphorylated Akt-positive patients, endocrine therapy has worse efficacy than in phosphorylated Akt-negative patients [42]. In this study, we found ER-α36 mediated tamoxifen-stimulated activation of Akt in cells with high levels of ER-α36 expression suggesting that the activation of the PI3K/Akt pathway mediated by ER-α36 contributes to the resistance and agonist action of tamoxifen.

The c-Myc protein is a nuclear transcription factor that plays an essential role in cell growth [43]. Previous studies have demonstrated that MAPK/ERK and PI3K/Akt pathways regulate c-Myc protein expression [44], [45], [46]. We found both E2 and tamoxifen induced c-Myc expression through ER-α36-mediated activation of ERK and Akt. Incubation of Hec1A cells with MEK inhibitor U0126 and PI3K inhibitor LY294002 blocked E2- and tamoxifen- induced c-Myc expression. Therefore, tamoxifen exerts agonist action through ER-α36-mediated non-genomic pathway.

In summary, we report here that ER-α36 is expressed on the plasma membrane and in the cytoplasm of endometrial carcinoma cells. We further demonstrated that both E2 and tamoxifen promoted proliferation of endometrial cancer cells through ER-α36-mediated activation of the MAPK/ERK and PI3K/Akt pathways and ER-α36 overexpression led to tamoxifen resistance in MCF-7 cells. Our results provide important novel information to further understand the molecular mechanisms underlying the agonist action of tamoxifen.

## Materials and Methods

### Materials and Reagents

All chemicals and reagents were purchased from Sigma unless otherwise indicated. Polyclonal anti-ERK1/2 antibody, polyclonal anti-phospho-ERK1/2 antibody (Thr^202^/Tyr^204^), polyclonal anti-caveolin-1 -TRITC antibody, monoclonal anti-c-Myc antibody, polyclonal anti-Akt antibody, monoclonal anti-ER-α66 (D-12) antibody and monoclonal anti-β-actin antibody were purchased from Santa Cruz Biotechnology (Santa Cruz, CA). Polyclonal anti-phospho-Akt (Ser^473^) antibody was obtained from Signalway Antibody (Pearland, TX). The ER-α36 specific antibody against the 20 unique amino acids at the C-terminal of ER-α36, ER-α36 expression plasmid, ER-α36 shRNA expression vector and the empty expression vector were described before [12], [14]. U0126 was purchased from Calbiochem (La Jolla, CA).

### Cell Culture and Cell Lines

MCF-7 human breast cancer cells were obtained from ATCC (Manassas, VA), and human Hec1A endometrial cancer cells were obtained from Dr. Li-Hui Wei (Peking University People's Hospital, Beijing). Both cell lines were maintained at 37°C with 5% CO_2_ in appropriate culture medium. To establish MCF-7 cells expressing recombinant ER-α36, cells were plated at a density of 1×10^5^ cells per 60-mm dish and transfected 24 hours later with an expression vector driven by the cytomagalovirus (CMV) promoter in the mammalian expression vector pCB6+ as described elsewhere [14], using the Lipofectamine 2000 (Invitrogen, Carlsbad, CA). The expression vector contains the full-length ER-α36 cDNA. The empty expression vector was also transfected into cells to serve as a control. Forty-eight hours after transfection, the cells were re-plated and selected with 500 µg/ml of G418 for two weeks. The medium was changed every three days until colonies appeared. Clones were expanded for further analysis. Clones with high ER-α36 expression were a mixture of more then twenty clones and termed MCF-7/ER-α36. A cell line with pooled clones transfected with the empty expression vector was named MCF-7/V and used as a control.

We also established cell lines from Hec1A cells transfected with an ER-α36 shRNA expression vector (Hec1A/RNAi) and the empty expression vector (Hec1A/V). Briefly, ER-α36 shRNA expression vector pRNAT-U6.1/Neo plasmid containing the shRNA against ER-a36 (GenScript Corp. TX) and the empty expression vector were transfected into Hec1A cells with Lipofectamine 2000 according to the manufacturer's instruction. Forty-eight hours after transfection, the cells were re-plated and selected with G418 (600 µg/ml) for two weeks. Clones were expanded for further analysis.

### Immunofluorescence and Confocal Microscopy

The cellular localization of protein was determined by indirect immunofluorescence. Hec1A or MCF-7 cells cultured on sterile glass coverslips were fixed in 4% paraformaldehyde in PBS for 10 min. After being permeabilized with 0.4% Triton X-100 at room temperature for 10 min, cells were blocked in 4% BSA-supplemented PBS for 1 h and incubated overnight at 4°C with anti-ER-α36-specific antibody. After three washes in PBS, the cells were labeled with FITC-conjugated secondary antibody. The DNA dye Hoechst 33258 was used for nuclear staining.

For double staining of ER-α36 and caveolin-1, after ER-α36 staining and wash in PBS, the cells were blocked in 4% BSA-supplemented PBS for 1 h at room temperature. After incubation with anti-caveolin-1-TRITC antibody overnight, the cells were further washed in PBS and stained with Hoechst 33258. Microscopic analyses were performed using a Confocal Laser-Scanning Microscope (Zeiss LSM 510 META, Germany).

### Semi-Quantitative RT-PCR

Total RNA was extracted by TRIzol reagent (Invitrogen, Carlsbad, CA) according to the manufacturer's instruction. Total RNA (1.6 µg) was used for production of the first strand cDNA by reverse transcriptase (Takara, Dalian, P.R.China). The following primer sets were designed for the amplification of human ER-α36 (BX640939, 1145–1434 bp): forward, 5′-CAAGTGGTTTCCTCGTGTCTAAAGC-3′ and reverse, 5′-TGTTGAGTGTTGGTTGC CAGG-3′; Human GAPDH mRNA was amplified by the forward primer 5′-ACGGATTTGG TCGTATTGGG-3′ and the reverse primer 5′-TGATTTTGGAGGGATCTCGC-3′.

### MTT Assay

Cell proliferation was analyzed using the 3-(4,5-dimethylthiazol-2-yl)-2,5- diphenyltetrazolium bromide (MTT) assay [47]. Briefly, cells were seeded in 96-well plates to a final concentration of 5×10^3^/well for MCF-7/V and MCF-7/ER36 cells or 3×10^3^/well for Hec1A/V and Hec1A/RNAi cells. MCF-7/V and MCF-7/ER36 cells were incubated in DMEM medium containing 10% FCS with the indicated treatments. Hec1A/V and Hec1A/RNAi cells were incubated in phenol-red free medium containing 2.5% dextran charcoal-stripped FCS (Biochrom AG, Berlin, Germany) with the indicated treatments. The cells were then incubated with MTT (0.5 mg/ml) for 4 h at 37°C. After removal the medium containing the MTT reagent, 150 µl of DMSO were added to each well. The plates were read at wavelength of 490 nm using a microplate reader (Bioteck Powerwave™, USA).

### Western Blotting Analysis

Cells were grown in phenol-red-free medium with 2.5% dextran charcoal- stripped FCS for 48–72 hours and then switched to medium without serum 12 h before stimulation by the agents indicated. The cells were collected in ice-cold PBS, and the cell extracts were prepared in RIPA buffer with the proteinase inhibitor cocktail from Sigma (St. Louis, MO). Cell lysates were boiled with gel-loading buffer for 5 min at 100°C, resolved on 10% SDS-PAGE, transferred to PVDF membranes, probed with appropriate antibodies and visualized with enhanced chemiluminescence (ECL) detection reagents (Amersham Pharmacia Biotech., Piscataway, NJ).

### Statistical Analysis

Statistical analysis was performed using paired-samples *t*-test, or ANOVA followed by the Student-Newman-Keuls testing to determine differences in means. *P*<0.05 was considered statistically significant.
